# Data on various allergen specific IgEs and prospective treatments on food-dependent exercise-induced anaphylaxis

**DOI:** 10.1016/j.dib.2018.04.097

**Published:** 2018-05-05

**Authors:** Norihide Murayama, Kikuno Murayama

**Affiliations:** Murayama Pediatrics, 3-2-33 Nagayoshi-Nagahara-Higashi Hirano-ku Osaka-shi, 547-0013 Osaka, Japan

**Keywords:** Multiple antigen positive, Food-dependent exercise-induced anaphylaxis (FDEIAn), Exercise-induced asthma (EIA), Leukotriene, Montelukast, Hydroxyzine

## Abstract

Food-dependent exercise-induced anaphylaxis (FDEIAn) is an anaphylactic reaction induced by physical exercise after ingestion of certain meals. FDEIAn is not very frequent, but recent case reports associated with various meals indicate an upward trend. Here, we report the data of various food specific IgEs and the clinical course of an experience with a patient who exhibited a unique FDEIAn reaction. Various food specific IgEs including staple food were positive with high levels. We could not find out the cause food of FDEIAn. Therefore we started preventive drug treatment. Specifically, only the skin symptoms (urticaria) were prevented by administering anti-histamine (hydroxyzine) daily, and respiratory symptoms (wheezing and distress) were prevented by daily administration of a leukotriene receptor antagonist (montelukast).

**Specifications Table**

**Table:** 

Subject area	Clinical Immunology
More specific subject area	Food-dependent exercise-induced anaphylaxis (FDEIAn)
Type of data	Table and Figure
How data was acquired	Clinical examination
Data format	Analyzed
Experimental factors	Efficacy of hydroxyzine and montelukast
Experimental feature	Treatment and mechanism of FDEIAn
Data source location	Osaka, Japan
Data accessibility	The data are supplied with this article

**Value of the data**

• We experienced a patient of food-dependent exercise-induced anaphylaxis.
• Most of His food specific IgEs including staple food were positive with high levels.
• This is the second report of combined anti-histamine and leukotriene receptor antagonist preventive treatment for food-dependent exercise-induced anaphylaxis.

## 1 Data

A case report of FDEIAn.

Patient: An 11-year-old male patient.

Body weight: 26.6 kg.

Height: 135.5 cm.

Patient history: No allergic disease history.

Family history: No food allergy and asthmatic history.

Present history; On June 7, 2005 patient exhibited exercise-induced total body urticaria and dyspnea 1 h after lunch at school and the symptoms disappeared after several hours. The following day, he and his mother visited our pediatric clinic for determination of the cause of symptoms.

## 2 Experimental design, materials, and methods

### 2.1 Condition following office visit

**Fig. 1:**
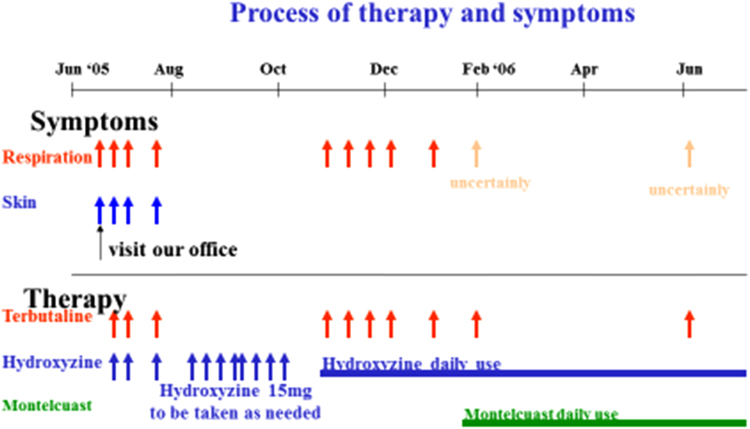
Clinical process.

One hour before the initial development of symptoms the patient ate a Japanese Medlar, which we considered was the source of his FDEIAn. We prescribed a tablet of terbutaline and 15 mg hydroxyzine powder to be administered as needed. Subsequently, the symptoms occurred without the patient consuming Japanese Medlar. In each case, the symptoms occurred within 90 min after meal ingestion following exercise. Furthermore, the frequency of FDEIAn development increased as became > 2–3 times every month (Fig. 1). Moreover, cereal radioallergosorbent test (RAST)s were > class 3, and Rice Cap RAST showed the highest level among the Cereal Cap RASTs. We considered that the cause of his FDEIAn was rice, which could not be excluded from his daily diet.

### 2.2 CAP-radioallergosorbent test (RAST, Table 1.)

The patient's clinical course caused us to suspect FDEIAn and, therefore, we performed various allergen tests using his peripheral blood and skin.

The RAST of inhaled Df (mite) antigen of was 33.40 UA/mL at the highest level. The cereal CAP-RAST of rice, wheat, and buckwheat were 12.90, 8.42, and 7.37 UA/mL respectively. The bean CAP-RAST of green peas, soya bean, corn, and sesame were 2.80, 6.63, 7.98, and 12.50 UA/mL respectively. The fruit CAP-RAST of orange, apple, peach, and banana were 4.16, 9.95, 3.33, and 5.57 UA/mL, respectively. The vegetable CAP-RAST of potato, sweet potato, and pumpkin were 4.46, 3.54, and 10.70 UA/mL respectively. The CAP-RAST of other foods: gelatin, egg white, crab, shrimp, blue mussel, tuna, and horse mackerel were > 0.34, > 0.34, 1.05, > 0.34, 0.74, and > 0.34 UA/mL respectively. The CAP-RAST results were mostly positive [1], especially those of food items, which were positive with high levels, and rice exhibited the highest level. Furthermore, the detection of so many positive food allergens is a very rare occurrence. We could not perform allergy test for Japanese Meddler because no RAST and prick test kits are available.

### 2.3 Hydroxyzine and montelukast effectively prevented FDEIAn (Fig. 1)

Daily administration of hydroxyzine was initiated from October 2005 for the prevention of anaphylaxis in this patient. Daily administration of hydroxyzine only inhibited the appearance of the urticaria but not the respiratory symptoms induced by exercise after meal ingestion (Fig. 1). Therefore, after a 2-month treatment with hydroxyzine, daily administration of montelukast was added to the regimen with the aim of avoiding airway contraction induced by exercise after food take. Oral administration of hydroxyzine and montelukast inhibited all the symptoms of FDEIAn in this patient.

### 2.4 Comparison of histamine and montelukast (Table 2)

**Fig. 2:**
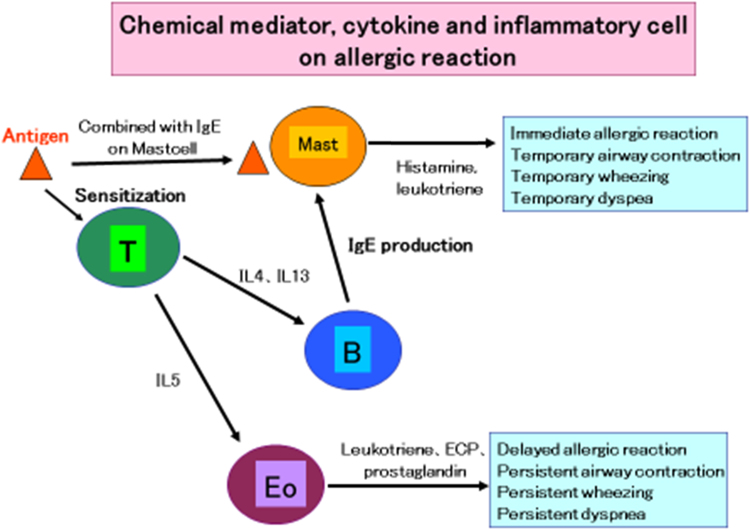
Chemical mediator, cytokine, and inflammatory cell in allergic reaction.

**Fig. 3:**
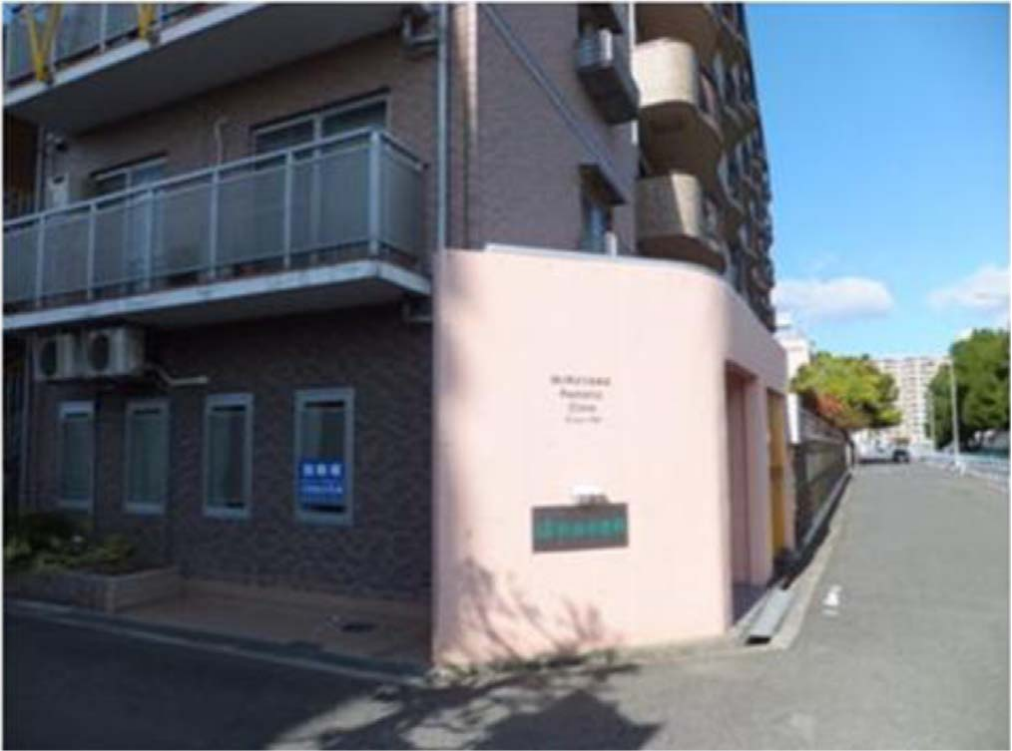
Murayama Pediatrics, established in 1994, Osaka, Japan.

**Fig. 4:**
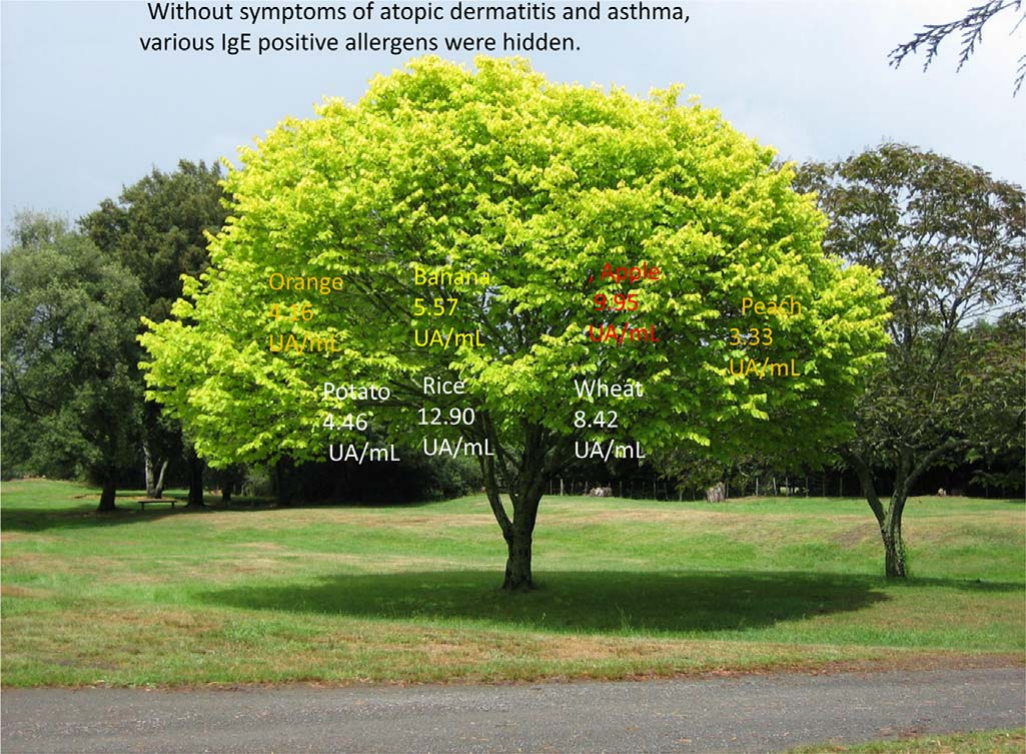
Three stages of anaphylactic response.

Mast cells [2] release histamine and leukotriene (Fig. 2). Although histamine has slight bronchoconstrictive activity, leukotriene [3], [4] has more than 1000 times the activity of histamine (Table 2). This fact suggests that montelukast treatment would be useful in the treatment of anaphylaxis with respiratory symptoms. This case report supports this notion (Fig. 3, Fig. 4).

**Table 1:** Allergen test result.

Total IgE (RIST)	1649 IU/mL
Peripheral blood	WBC 5900/mcL, Eo 6.3%
RAST Inhaled Antigen	① Df (mite) 33.40 UA/mL
RAST Cereal	② Rice 12.90 UA/mL, ⑥ Wheat 8.42 UA/mL, ⑦ Buckwheat 7.37 UA/mL
RAST Bean	Green peas 2.80 UA/mL, ⑧ Soya bean 6.63 UA/mL, Corn 7.98 UA/mL, ③Sesame 12.50 UA/mL
RAST Fruits	Orange 4.16 UA/mL, ⑤Apple 9.95 UA/mL, Peach 3.33 UA/mL, Banana 5.57 UA/mL
RAST Vegetable	Potato 4.46 UA/mL, Sweet Potato 3.54 UA/mL, ④Pumpkin 10.70 UA/mL
RAST Other food	Gelatin > 0.34 UA/mL, Egg white > 0.34 UA/mL, Crab 1.05 UA/mL, Shrimp > 0.34 UA/mL, Blue mussel 0.74 UA/mL, Tuna > 0.34 UA/mL, horse mackerel > 0.34 UA/mL
Prick test	Egg white, milk, wheat, soya bean, and sesame were all negative.
	[Number is according to a high degree of RAST(UA/mL)]

**Table 2:** Comparisons of histamine and leukotriene.

	Histamine	Leukotriene
Producing cells	Mast cells, lung, liver, gastric mucosa	Mast, eosinophil, and other cells
Bronchoconstrictive action	Slight	> 1000 times that of histamine
Main action	Fall in blood pressure, increased vascular permeability, smooth muscle contraction, vascular dilatation, increased glandular secretion	Bronchoconstriction, increased respiratory reactivity by eosinophil cell action, increased Th2 cytokine expression and production
